# Classification and prediction of Alzheimer’s disease stages and conversion from mild cognitive impairment based on multimodal data fusion

**DOI:** 10.3389/fnagi.2026.1802842

**Published:** 2026-04-29

**Authors:** Yao Wang, Haijun Li, Yadong Wang, Yingying Wang, Zan Dong, Yiying Lu

**Affiliations:** 1 Department of Geriatric, Taizhou Central Hospital (Taizhou University Hospital), Taizhou, Zhejiang, China; 2 Department of Neurology and Geriatrics, Taizhou Hospital of Traditional Chinese Medicine, Taizhou, Zhejiang, China

**Keywords:** Alzheimer’s disease, conversion prediction, disease staging, mild cognitive impairment, multimodal data fusion

## Abstract

**Objective:**

This study aimed to develop multimodal prediction models based on real-world clinical data for classifying different stages of Alzheimer’s disease (AD) and for predicting the conversion from mild cognitive impairment (MCI) to AD.

**Methods:**

A single-center retrospective real-world cohort study was conducted. A total of 658 individuals aged ≥50 years were included and classified into cognitively normal (CN), MCI, and AD groups. Demographic characteristics, neurocognitive assessment results, conventional magnetic resonance imaging (MRI) features, and blood-based biomarkers were collected. Logistic regression was used to construct pairwise classification models for disease stages and prediction models for MCI-to-AD conversion. Model performance was evaluated through stepwise integration of multimodal features. Discrimination ability was assessed using the area under the receiver operating characteristic curve (AUC), together with calibration curves and decision curve analysis. In a sub-cohort with thin-slice MRI data, the additional value of hippocampal volume was further examined.

**Results:**

Significant differences were observed among disease stages in cognitive function, imaging markers, and blood biomarkers (all *p* < 0.05). Multimodal fusion models achieved the best performance in disease stage classification (CN vs. AD: AUC = 0.96 ± 0.01; MCI vs. AD: AUC = 0.86 ± 0.03). The conversion prediction model integrating multimodal features showed good discrimination (AUC = 0.87) and satisfactory calibration. In the thin-slice MRI sub-cohort, inclusion of hippocampal volume increased the AUC to 0.88.

**Conclusion:**

Multimodal prediction models based on real-world clinical data improved the accuracy of AD stage classification and the prediction of MCI-to-AD conversion risk. These models demonstrated good clinical feasibility. High-resolution structural imaging markers further enhanced predictive performance in selected populations.

## 1 Introduction

Alzheimer’s disease (AD) is one of the most common neurodegenerative disorders. It is characterized by progressive cognitive decline and impairment in activities of daily living (Jack et al., 2024). With the acceleration of global population aging, the burden of AD and related cognitive disorders continues to increase. AD has become a major public health issue affecting older adults and healthcare systems worldwide (Li et al., 2021). Although advances have been made in understanding disease mechanisms and diagnostic techniques, effective disease-modifying treatments for AD remain limited. Early identification and risk assessment are therefore considered key strategies for delaying disease progression and improving patient management (Cummings et al., 2024). Mild cognitive impairment (MCI) is widely regarded as an intermediate stage between normal aging and dementia. A proportion of individuals with MCI progress to AD during follow-up (Raimo et al., 2024). However, the MCI population shows marked heterogeneity in clinical manifestations and biological characteristics. Risk assessment based solely on clinical symptoms or a single examination marker is often insufficient to accurately determine individual conversion risk (Thomas et al., 2022). Therefore, identifying patients with a high risk of conversion during the MCI stage has become one of the key issues in current AD research and clinical practice.

Previous studies have shown that AD development involves multi-level alterations, including cognitive impairment, structural brain atrophy, and amyloid and tau-related pathological changes (Hazan et al., 2024). In structural imaging studies, brain atrophy is considered an important imaging feature of AD. Among these features, hippocampal atrophy is widely regarded as the most representative early imaging marker, and its volume reduction is closely associated with cognitive decline. Previous studies have applied hippocampal atrophy characteristics together with machine learning methods to achieve automatic diagnosis of AD, and good classification performance has been reported (Uysal and Ozturk, 2020). In addition to the hippocampus, structures in the medial temporal lobe, changes in cortical thickness, and structural alterations in other brain regions can also reflect disease stage information. Joint analysis of structural information from multiple brain regions can further improve the identification ability at the MCI stage (Uysal and Ozturk, 2024). In recent years, with the development of machine learning and deep learning techniques, automated analysis based on brain MRI has gradually become an important direction in AD research. Some studies extracted structural features from MRI using deep feature fusion networks or deep learning models to perform automatic classification of MCI and AD and to evaluate disease severity (Illakiya et al., 2024; Singh and Kumar, 2024; Ahmadi et al., 2024). However, many of these studies relied on high-resolution imaging data or complex deep learning models, and their application in real-world clinical settings still faces certain challenges.

On the other hand, multimodal research strategies have attracted increasing attention. Integration of cognitive assessments, neuroimaging findings, and biomarker information may provide a more comprehensive characterization of disease status. Existing evidence indicates that multimodal models outperform single-modality approaches in AD stage classification and conversion prediction (Lee et al., 2024). Nevertheless, several limitations remain. First, some studies rely heavily on cerebrospinal fluid analysis or molecular imaging, which limits applicability in routine clinical settings. Second, many models are derived from highly selected research cohorts that differ from real-world clinical populations (Galvin et al., 2024). In clinical practice, available examinations are influenced by medical resources, patient compliance, and clinical decision-making. High-precision imaging or invasive tests are not accessible for all patients. Therefore, prediction models based on routinely available data are essential to improve clinical feasibility. In addition, evaluating the incremental value of high-resolution structural imaging markers in selected patients may support stratified and individualized assessment strategies.

Based on these considerations, this retrospective real-world cohort study integrated demographic data, neurocognitive assessments, conventional MRI markers, and blood-based biomarkers to construct multimodal prediction models for AD stage classification. The performance of these models in predicting MCI-to-AD conversion was further evaluated. In a sub-cohort with thin-slice MRI data, the additional contribution of hippocampal volume and other high-resolution structural markers was explored. The main contributions of this study included: (1) constructing an AD disease stage classification model based on real-world multimodal clinical data; (2) evaluating the ability to predict the conversion from MCI to AD; and (3) further analyzing the additional contribution of structural imaging indicators, such as hippocampal volume, to the predictive performance of the model in a thin-slice MRI sub-cohort. This study aimed to provide a feasible multimodal modeling strategy for AD stage identification and risk evaluation of MCI conversion in real-world clinical environments.

## 2 Methods

### 2.1 Study design and participants

**Figure 1:**
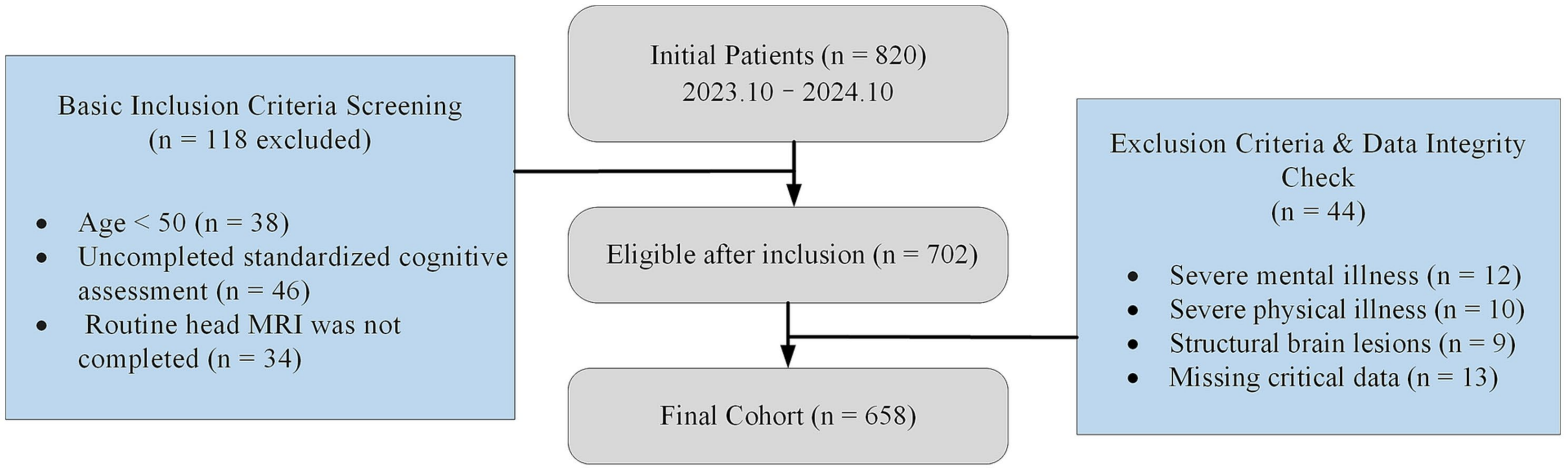
Flowchart of participant selection.

A single-center retrospective real-world cohort design was used. Participants were consecutively recruited from the outpatient and inpatient departments of Neurology and Geriatrics at Taizhou Hospital of Traditional Chinese Medicine between October 2023 and October 2024. A total of 658 patients who met the inclusion criteria were enrolled. All data were derived from routine clinical records, including demographic characteristics, medical history, neuroimaging examinations, and cognitive assessments performed during standard clinical care. Laboratory results were obtained according to tests conducted in routine diagnostic practice. No additional examinations or research-specific interventions were introduced, ensuring that the dataset reflected real-world clinical practice rather than a controlled research database. Inclusion criteria were as follows: (1) age ≥50 years; (2) completion of at least one standardized cognitive assessment; (3) completion of conventional brain MRI; (4) availability of complete baseline clinical and imaging data; (5) a clearly defined baseline diagnostic stage (cognitively normal [CN], MCI, or AD). Exclusion criteria included: (1) severe psychiatric disorders such as schizophrenia or bipolar disorder); (2) severe systemic diseases that could interfere with cognitive assessment, including severe infections or end-stage malignancies; (3) major structural brain lesions that affected image interpretation, such as large brain tumors or sequelae of severe traumatic brain injury; (4) extensive missing clinical data precluding statistical analysis. The study protocol was reviewed and approved by the Medical Ethics Committee of Taizhou Hospital of Traditional Chinese Medicine (Approval No. LL2026-LW-001). Given the retrospective nature and absence of additional interventions, informed consent was waived. The study was conducted in accordance with the Declaration of Helsinki and relevant ethical guidelines. The participant selection process is shown in Figure 1.

### 2.2 Baseline diagnostic classification and follow-up outcome definition

#### 2.2.1 Baseline diagnostic classification

Baseline diagnoses were independently determined by two neurologists with senior professional titles based on current clinical consensus criteria. Medical history, neurological examination findings, cognitive test results, and imaging features were comprehensively reviewed. Disagreements were resolved through discussion. This process was conducted retrospectively. Participants were classified as CN, MCI, or AD according to established national and international diagnostic criteria.

#### 2.2.2 Follow-up and conversion outcome

A retrospective follow-up was performed for participants whose baseline diagnosis was MCI. The follow-up began at the time of the first confirmed diagnosis of MCI. The end point of follow-up was defined as the time of the last outpatient or inpatient follow-up record, or the time when AD was clearly diagnosed. The follow-up duration ranged from 3 to 12 months. The median follow-up time was 9 months (interquartile range: 6–11 months). Follow-up information was obtained from outpatient revisit records and the electronic medical record system. During follow-up, the conversion from MCI to AD was judged by two neurologists with the professional title of associate chief physician or higher based on the available clinical information. The diagnostic criteria referred to the Alzheimer’s disease guidelines proposed by the National Institute on Aging–Alzheimer’s Association (NIA-AA). The final diagnosis was determined by combining patient history, results of cognitive scale assessments, and imaging findings. Participants whose follow-up outcome could not be clearly determined were excluded from the conversion prediction analysis. All included participants had clear follow-up outcomes, and no loss to follow-up occurred. Outcomes were defined as follows: Non-conversion group: MCI diagnosis maintained during follow-up; Conversion group: progression from MCI to AD during follow-up. Follow-up duration was recorded in months for each participant, along with conversion status.

### 2.3 Collection of clinical and demographic data

Baseline demographic and clinical data were extracted from electronic medical records. Variables included age, sex, years of education, source of visit (outpatient or inpatient), marital status or living arrangement, comorbidities (hypertension, diabetes, hyperlipidemia, coronary heart disease, other cardiovascular diseases, and prior stroke), and history of depression or anxiety. These variables were considered potential confounders or candidate predictors in subsequent analyses.

### 2.4 Imaging data acquisition and processing

#### 2.4.1 Conventional magnetic resonance imaging

All participants underwent conventional brain MRI, including axial T1-weighted imaging (T1WI), axial T2-weighted imaging (T2WI), and fluid-attenuated inversion recovery (FLAIR) sequences. Semi-quantitative imaging markers were derived to reflect neurodegeneration and cerebrovascular burden. These included medial temporal lobe atrophy (MTA) scores, Fazekas scores (WMH) for white matter hyperintensities, and the presence of cerebral infarction or other vascular lesions. Imaging assessments were independently performed by two experienced neuroradiologists who were blinded to clinical diagnoses. Discrepancies were resolved by consensus.

#### 2.4.2 Hippocampal volume measurement

A subset of participants underwent thin-slice MRI for clinical reasons and had hippocampal volume measurements available. Hippocampal volume was recorded for these participants only. As thin-slice MRI was not routinely available for all patients in real-world practice, hippocampal volume was included exclusively in sub-cohort analyses to evaluate its incremental predictive value. It was not considered a mandatory variable for the full cohort.

### 2.5 Cognitive function assessment

All participants completed standardized neurocognitive assessments during routine clinical care. These included: (1) Mini-Mental State Examination (MMSE) (Galea and Woodward, 2005), with a total score of 30 points. This test assessed orientation, memory, attention, calculation, language, and visuospatial abilities. Lower scores indicated greater global cognitive impairment. (2) Montreal Cognitive Assessment (MoCA) (Islam et al., 2023), also scored out of 30 points, covering executive function, attention, memory, language, visuospatial ability, and orientation. Lower scores reflected broader cognitive decline. One point was added for participants with ≤12 years of education. (3) Delayed recall test (De Simone et al., 2019), based on a word learning–delayed recall paradigm with a maximum score of 10 points. The number of correctly recalled words during the delay phase was recorded. Lower scores indicated greater impairment in memory storage and retrieval. All cognitive assessments were conducted by trained professionals and extracted from existing clinical records.

### 2.6 Biomarker measurements

Peripheral venous blood samples were collected to measure the following biomarkers: A*β*42, Aβ40 or the Aβ42/Aβ40 ratio, total tau, phosphorylated tau (*p*-Tau), and apolipoprotein E ε4 (APOE ε4) carrier status (carrier vs. non-carrier). Blood-based biomarkers were selected to enhance data availability and model applicability in real-world clinical settings.

### 2.7 Statistical analysis and model construction

#### 2.7.1 Pairwise classification models for disease stages

Logistic regression-based models were used to evaluate discrimination among different AD stages. During model construction, features from different modalities were used, including demographic and basic clinical variables, neurocognitive scale scores, imaging indicators, and blood biomarkers. Before modeling, these variables were organized and combined to form a joint feature vector $X=(x_{1},x_{2},\ldots ,x_{p})$ , where *p* represents the total number of features included in the model, and $x_{i}$ denotes the value of the *i*-th feature variable. The combined feature vector was then entered into a Logistic regression model for analysis. This approach belongs to feature-level multimodal fusion, in which features from different sources are integrated to make use of multimodal information. For the classification of disease stages, a pairwise classification strategy was applied. Three binary prediction models were established: (1) CN group vs. MCI group; (2) CN group vs. AD group; and (3) MCI group vs. AD group. For each binary classification task, the Logistic regression model was defined as:


$$P(Y=1|X)=\frac{1}{1+\exp[-(\beta_{0}+\sum_{i=1}^{p}\beta_{i}x_{i})]}$$

In this formula, *Y* represents the binary outcome variable. In the analysis of CN vs. MCI, CN was coded as 0 and MCI as 1. In the comparison of CN vs. AD, CN was coded as 0 and AD as 1. In the analysis of MCI vs. AD, MCI was coded as 0 and AD as 1. The parameter $\beta_{0}$ denotes the intercept of the model, and $\beta_{i}$ represents the regression coefficient of the *i*-th feature. These coefficients indicate the contribution of each feature to the classification result. Model parameters were estimated using maximum likelihood estimation, which allowed the relative importance of each feature in disease stage classification to be determined.

Predictor variables included demographic and basic clinical features, imaging markers, cognitive test scores, and blood biomarkers. Models with single-modality features and multimodal combinations were constructed stepwise to compare predictive performance across data sources.

#### 2.7.2 Prediction models for MCI-to-AD conversion

For participants with MCI, conversion to AD during follow-up was defined as the outcome variable (non-conversion = 0; conversion = 1). The model used the same Logistic regression framework as that applied in the disease stage classification analysis. The input variables were also integrated from different modalities to form a joint feature vector. Time-invariant covariates, including age, sex, education level, visit source, major comorbidities, the presence of cerebral infarction or other vascular lesions and APOE ε4 status, were included to control for confounding. Baseline cognitive scores, imaging markers, and blood biomarkers were treated as candidate predictors. The prediction model developed in this study was used to estimate the risk of conversion from MCI to AD during approximately 1 year of follow-up. Logistic regression was applied to assess the predictive value of different feature combinations. Five models were constructed using a stepwise integration strategy:

Model 1: demographic and basic clinical variables only;

Model 2: Model 1 plus cognitive assessments (MMSE, MoCA, delayed recall);

Model 3: Model 2 plus conventional imaging markers (MTA and WMH);

Model 4: Model 3 plus blood biomarkers (Aβ42, Aβ40, Aβ42/Aβ40, tau, p-Tau);

Model 5: thin-slice MRI extension model, developed only in the sub-cohort with thin-slice MRI, adding hippocampal volume to Model 4. By comparing the predictive performance of different models, the incremental value of multimodal information in predicting the risk of conversion from MCI to AD could be systematically evaluated.

#### 2.7.3 Thin-slice MRI sub-cohort analysis

Analyses involving hippocampal volume were restricted to participants with available thin-slice MRI data. Hippocampal volume was added to the main model to compare performance changes and to evaluate the incremental value of high-resolution structural markers in specific populations.

#### 2.7.4 Model development, internal validation, and performance evaluation

To assess the stability and predictive performance of the model, five-fold cross-validation was used for internal validation in this study. Specifically, the dataset was randomly divided into five subsets, and the outcome distribution of each subset was kept as similar as possible. In each round, four subsets were used to train the model, and the remaining subset served as the validation set. This procedure was repeated five times so that every subset was used once for validation. The final model performance was summarized by the mean values of the evaluation metrics across the five folds. Model discrimination was primarily evaluated using the area under the receiver operating characteristic curve (AUC). Sensitivity, specificity, and Brier score were also reported to assess classification performance and prediction error. Accuracy was additionally reported for disease stage classification analyses. Calibration curves were used to evaluate agreement between predicted probabilities and observed outcomes. Bootstrap resampling was applied to obtain bias-corrected calibration curves. Bootstrap resampling was performed with 1,000 iterations. Decision curve analysis (DCA) was applied to estimate the net benefit of the model across different threshold probabilities. The cross-validation results were also considered to examine the clinical stability of the model during internal validation.

#### 2.7.5 Statistical analysis

Continuous variables were expressed as mean ± standard deviation and standardized when necessary. Categorical variables were presented as counts and percentages. Candidate variables were screened based on clinical relevance and statistical considerations to reduce multicollinearity and overfitting. Candidate variables were determined in advance based on previous studies on the prediction of MCI conversion to AD and their clinical relevance (Agostinho et al., 2024; Valizadeh et al., 2025). All analyses were performed using R software (version 4.0.0). Logistic regression and performance evaluation were implemented using relevant R functions and packages, including pROC. All tests were two-sided, and *p* < 0.05 was considered statistically significant.

Participants without definitive conversion outcomes were excluded from conversion analyses. For missing predictor data with low missing rates, complete-case analysis was applied. Examinations not routinely available in real-world practice, such as blood biomarkers and thin-slice MRI, were analyzed only in participants with corresponding data, without imputation.

## 3 Results

### 3.1 Baseline characteristics of participants

Table 1 summarizes the demographic and baseline clinical characteristics of the 658 participants. Significant differences were observed among the three groups in age, years of education, and visit source (*p* < 0.05). Sex distribution and most cardiometabolic comorbidities were comparable across groups (*p* > 0.05), indicating overall good baseline comparability.

**Table 1:** Comparison of baseline demographic characteristics, clinical features, and imaging data among the three groups (*n* = 658).

Variable	CN (*n* = 210)	MCI (*n* = 300)	AD (*n* = 148)	*p* value
Demographic and visit characteristics				
Age, years, mean ± SD	66.6 ± 6.8	71.1 ± 6.3	74.2 ± 6.7	<0.001
Female, *n* (%)	114 (54.3)	160 (53.3)	86 (58.1)	0.74
Years of education, mean ± SD	12.2 ± 3.1	10.4 ± 3.8	9.6 ± 3.5	<0.001
Inpatient visit, *n* (%)	46 (21.9)	108 (36.0)	64 (43.2)	<0.001
Living alone, *n* (%)	38 (18.1)	98 (32.7)	46 (31.1)	0.002
Clinical history and comorbidities				
Hypertension, *n* (%)	92 (43.8)	168 (56.0)	82 (55.4)	0.040
Diabetes mellitus, *n* (%)	40 (19.0)	66 (22.0)	34 (23.0)	0.55
Hyperlipidemia, *n* (%)	70 (33.3)	110 (36.7)	48 (32.4)	0.79
Coronary heart disease or cardiovascular disease, *n* (%)	28 (13.3)	52 (17.3)	26 (17.6)	0.46
History of stroke, *n* (%)	22 (10.5)	36 (12.0)	24 (16.2)	0.19
History of depression or anxiety, *n* (%)	28 (13.3)	70 (23.3)	28 (18.9)	0.23
Imaging data				
Conventional MRI (T1/T2/FLAIR), *n* (%)	210 (100)	300 (100)	148 (100)	—
Thin-slice MRI with hippocampal volume, *n* (%)	42 (20.0)	84 (28.0)	56 (37.8)	<0.001
Presence of cerebral infarction or vascular lesions, *n* (%)	20 (9.5%)	41 (1.7%)	41 (2.8%)	—

### 3.2 Cognitive function, imaging findings, and blood biomarkers across disease stages

Comparisons of cognitive assessments, imaging markers, and blood-based biomarkers among different disease stages are shown in Table 2. For cognitive performance, significant differences were observed among the three groups in MMSE, MoCA, and delayed recall scores (all *p* < 0.001). Regarding imaging markers, both MTA scores and WMH for white matter hyperintensities differed significantly across groups (all *p* < 0.001). Blood biomarker analysis showed significant differences in Aβ42 levels and the Aβ42/Aβ40 ratio among the three groups. These markers gradually decreased with disease progression (all *p* < 0.001). Aβ40 levels showed smaller between-group differences but remained statistically significant (*p* = 0.014). Tau and phosphorylated tau (*p*-Tau) concentrations were highest in the AD group, intermediate in the MCI group, and lowest in the CN group (all *p* < 0.001).

**Table 2:** Comparison of cognitive function, imaging markers, and blood biomarkers across disease stages (mean ± SD, *n* = 658).

Variable	CN (*n* = 210)	MCI (*n* = 300)	AD (*n* = 148)	*p* value
Cognitive assessments				
MMSE, total score	28.4 ± 1.1	25.2 ± 3.1	18.8 ± 3.3	<0.001
MoCA, total score	26.4 ± 1.6	21.1 ± 4.3	15.0 ± 4.3	<0.001
Delayed recall	7.4 ± 1.5	5.6 ± 2.3	1.8 ± 1.2	<0.001
Imaging markers				
MTA score	0.89 ± 0.54	1.52 ± 0.67	2.46 ± 0.76	<0.001
WMH	1.15 ± 0.64	1.30 ± 0.73	2.05 ± 0.80	<0.001
Blood biomarkers				
Aβ42 (pg/mL)	33.1 ± 7.9	29.9 ± 7.7	27.8 ± 7.1	<0.001
Aβ40 (pg/mL)	430 ± 69	436 ± 89	441 ± 85	0.014
Aβ42/Aβ40 ratio	0.077 ± 0.013	0.069 ± 0.012	0.063 ± 0.010	<0.001
Tau (pg/mL)	2.38 ± 0.74	2.79 ± 0.79	3.45 ± 0.90	<0.001
p-Tau (pg/mL)	0.86 ± 0.30	1.08 ± 0.31	1.35 ± 0.41	<0.001

Cognitive assessments and imaging markers were analyzed in the full cohort. Blood biomarker analyses were conducted in participants with available test results, and sample sizes varied across biomarkers.

### 3.3 Predictive performance of pairwise classification models for disease stages

The predictive performance of pairwise classification models across disease stages in Table 3. Model 1, which included only demographic and basic clinical features, showed limited discrimination ability (AUC range: 0.65–0.74). As cognitive scores, imaging markers, and blood biomarkers were sequentially added, model performance improved consistently, with AUC values increasing to 0.86–0.96 in the fully integrated models. Across all classification tasks, Model 4 integrating multimodal features achieved the best performance. The highest discrimination was observed in the CN versus AD task (AUC = 0.96 ± 0.01), whereas classification between MCI and AD showed greater difficulty (AUC = 0.86 ± 0.03).

**Table 3:** Predictive performance of pairwise classification models for different disease stages under five-fold cross-validation (mean ± SD, *n* = 658).

Classification task	Model	AUC	Accuracy	Sensitivity	Specificity
CN vs. MCI	Model 1	0.69 ± 0.04	0.66 ± 0.04	0.63 ± 0.05	0.69 ± 0.05
	Model 2	0.83 ± 0.03	0.79 ± 0.05	0.81 ± 0.02	0.77 ± 0.04
	Model 3	0.88 ± 0.02	0.84 ± 0.03	0.86 ± 0.05	0.82 ± 0.02
	Model 4	0.91 ± 0.01	0.87 ± 0.05	0.89 ± 0.04	0.85 ± 0.04
CN vs. AD	Model 1	0.75 ± 0.04	0.72 ± 0.04	0.74 ± 0.02	0.70 ± 0.05
	Model 2	0.92 ± 0.05	0.89 ± 0.03	0.91 ± 0.01	0.87 ± 0.02
	Model 3	0.95 ± 0.02	0.92 ± 0.04	0.94 ± 0.03	0.90 ± 0.01
	Model 4	0.96 ± 0.01	0.94 ± 0.03	0.95 ± 0.02	0.92 ± 0.02
MCI vs. AD	Model 1	0.66 ± 0.04	0.63 ± 0.05	0.61 ± 0.05	0.65 ± 0.05
	Model 2	0.79 ± 0.03	0.75 ± 0.02	0.77 ± 0.06	0.73 ± 0.03
	Model 3	0.84 ± 0.02	0.80 ± 0.05	0.82 ± 0.03	0.78 ± 0.04
	Model 4	0.87 ± 0.04	0.83 ± 0.04	0.85 ± 0.02	0.81 ± 0.03

In addition, the results of five-fold cross-validation showed that the AUC values of the classification models ranged from 0.66 to 0.96, with standard deviations between 0.01 and 0.05. These findings indicate that the models maintained relatively stable discrimination under different data partitions.

### 3.4 Baseline characteristics of MCI conversion and non-conversion groups

Among the 300 participants with a baseline diagnosis of MCI, the median follow-up time was 9 months (interquartile range: 6–11 months). During follow-up, 80 patients (26.7%) progressed to Alzheimer’s disease, while the remaining 220 patients (73.3%) did not develop conversion during the observation period.

At baseline, the conversion group already showed unfavorable features across multiple domains. Compared with the non-conversion group, the conversion group had significantly lower global cognitive performance. Differences in MMSE and MoCA scores were statistically significant (both *p* < 0.001), indicating more pronounced cognitive impairment at the MCI stage. Imaging analyses showed more severe structural brain changes in the conversion group. This was mainly reflected by higher MTA scores (*p* = 0.001) and increased white matter lesion burden (*p* < 0.05). At the biological level, the conversion group showed a lower Aβ42/Aβ40 ratio and higher tau and p-Tau levels (all *p* < 0.05). These findings suggested the presence of AD-related pathological changes. Differences in Aβ42 and Aβ40 alone were not statistically significant. Overall, impaired cognition, structural brain alterations, and tau-related biomarker abnormalities jointly indicated a higher risk of progression to AD during the MCI stage. These features provided key candidates for subsequent prediction modeling (Table 4).

**Table 4:** Baseline characteristics of MCI conversion and non-conversion groups [mean ± SD, *n* (%), *n* = 300].

Variable	Conversion (*n* = 80)	Non-conversion (*n* = 220)	*p* value
Demographic characteristics	—	—	—
Age, years	73.8 ± 6.4	70.2 ± 6.0	0.021
Female	42 (52.5)	118 (53.6)	0.89
Years of education	9.2 ± 3.8	10.9 ± 3.7	0.032
Inpatient visit	36 (45.0)	72 (32.7)	0.047
Living alone	30 (37.5)	68 (30.9)	0.32
Clinical history and comorbidities	—	—	—
Hypertension	54 (67.5)	114 (51.8)	0.022
Diabetes mellitus	22 (27.5)	44 (20.0)	0.18
Hyperlipidemia	34 (42.5)	76 (34.5)	0.26
Coronary heart disease	14 (17.5)	38 (17.3)	0.93
History of stroke	12 (15.0)	24 (10.9)	0.32
History of depression or anxiety	18 (22.5)	52 (23.6)	0.87
Cognitive assessments	—	—	—
MMSE, total score	23.1 ± 3.6	26.0 ± 2.6	<0.001
MoCA, total score	18.3 ± 4.5	22.1 ± 3.7	<0.001
Delayed recall	4.1 ± 2.4	6.2 ± 2.1	<0.001
Imaging markers	—	—	—
MTA score	1.90 ± 0.67	1.37 ± 0.61	0.001
WMH	1.61 ± 0.64	1.18 ± 0.72	0.03
Blood biomarkers	—	—	—
Aβ42 (pg/mL)	28.1 ± 8.2	30.5 ± 7.4	0.12
Aβ40 (pg/mL)	442 ± 88	434 ± 89	0.11
Aβ42/Aβ40 ratio	0.064 ± 0.014	0.071 ± 0.011	0.021
Tau (pg/mL)	2.92 ± 0.85	2.74 ± 0.77	0.018
p-Tau (pg/mL)	1.22 ± 0.29	1.02 ± 0.30	0.003
APOE ε4 carrier	42 (52.5)	84 (38.2)	0.016

### 3.5 Performance of prediction models for MCI-to-AD conversion

Prediction performance improved progressively with the inclusion of additional feature sets. Compared with the model including only demographic and basic clinical features (AUC = 0.67), discrimination increased after adding cognitive scores (AUC = 0.79). Further inclusion of imaging markers led to additional improvement (AUC = 0.84). The full-feature model achieved the best performance (AUC = 0.87) and showed the lowest prediction error (Brier score = 0.12). (Table 5).

**Table 5:** Predictive performance of models with different feature combinations for MCI-to-AD conversion under five-fold cross-validation.

Model	AUC	Sensitivity	Specificity	Brier score
Model 1	0.67 ± 0.04	0.63 ± 0.02	0.70 ± 0.05	0.20 ± 0.03
Model 2	0.79 ± 0.03	0.75 ± 0.03	0.77 ± 0.02	0.16 ± 0.01
Model 3	0.84 ± 0.04	0.80 ± 0.05	0.82 ± 0.04	0.14 ± 0.03
Model 4	0.87 ± 0.02	0.84 ± 0.03	0.85 ± 0.03	0.12 ± 0.01

The five-fold cross-validation results also showed that the standard deviations of the AUC values for all models were within a small range (0.02–0.04), suggesting that the models had good stability under different data splits.

To further examine the influence of each predictor on the risk of conversion from MCI to AD, key variables were selected during multivariable model construction based on the results of univariable analysis and their clinical relevance. These variables were then included in a multivariable Logistic regression analysis to quantitatively estimate the contribution of each feature to the prediction outcome. The results indicated that older age, higher MTA score, elevated Tau level, and APOEε4 carrier status were significantly associated with an increased risk of conversion from MCI to AD. In contrast, longer years of education, higher MMSE score, and a higher Aβ42/Aβ40 ratio were significantly related to a lower conversion risk. Among these variables, a lower MMSE score (OR = 0.80, 95% CI: 0.72–0.89) and a higher MTA score (OR = 1.76, 95% CI: 1.21–2.56) showed significant associations with conversion risk. (Table 6).

**Table 6:** Multivariable logistic regression analysis of predictors for MCI-to-AD conversion.

Variable	OR	95% CI	*p* value
Age (per 1-year increase)	1.08	1.01–1.15	0.024
Years of education (per 1-year increase)	0.91	0.84–0.99	0.031
MMSE score (per 1-point increase)	0.80	0.72–0.89	<0.001
MTA score (per 1-point increase)	1.76	1.21–2.56	0.003
Aβ42/Aβ40 ratio (per 0.01 increase)	0.74	0.58–0.95	0.018
Tau (per 1 pg./mL increase)	1.42	1.11–1.82	0.006
APOE ε4 carrier (yes vs. no)	1.93	1.08–3.46	0.027

Continuous variables were included in the model according to unit change; APOE ε4 was treated as a binary variable.

### 3.6 Calibration performance and decision curve analysis for MCI-to-AD conversion models

**Figure 2:**
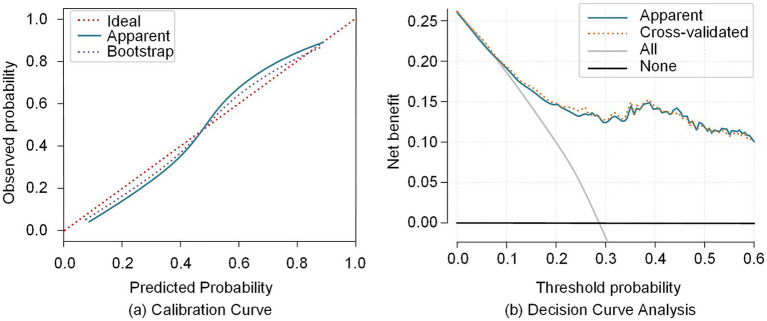
Calibration curve and decision curve analysis of the full model for predicting conversion from MCI to AD. **(a)** Calibration curve illustrating the agreement between predicted and observed probabilities. **(b)** Decision curve analysis showing the net benefit across different threshold probabilities.

The calibration plot showed that the predicted probabilities of the full model were generally consistent with the observed conversion rates. The bias-corrected calibration curve closely followed the apparent curve, indicating satisfactory calibration and model stability (Figure 2A).

Decision curve analysis demonstrated that within a threshold probability range of 0.10–0.60, the full-feature model consistently achieved higher net benefit than the “treat-all” and “treat-none” strategies. This finding suggested potential clinical utility within acceptable decision thresholds. Further comparison between apparent and cross-validated decision curves showed similar trends, indicating stable internal validation and a low risk of overfitting (Figure 2B).

### 3.7 Thin-slice MRI sub-cohort analysis

To further evaluate the incremental value of high-resolution structural imaging markers in predicting conversion from MCI to AD, a sub-cohort analysis was conducted in participants with available thin-slice MRI data. A total of 182 participants who completed thin-slice MRI scanning were included in this sub-cohort. Among them, 84 patients were diagnosed with MCI at baseline and were used for subsequent conversion prediction analysis. The baseline characteristics and hippocampal volume distribution of the thin-slice MRI sub-cohort (*n* = 182) are presented in Table 7. The overall pattern showed that as the disease stage progressed from CN to MCI and AD, cognitive scores gradually decreased. At the same time, hippocampal volume differed significantly across disease stages (*p* = 0.002).

**Table 7:** Baseline characteristics and hippocampal volume comparison among different disease stages in the thin-slice MRI sub-cohort (*n* = 182).

Variable	CN (*n* = 42)	MCI (*n* = 84)	AD (*n* = 56)	*p* value
Age (years), mean ± SD	66.8 ± 6.5	71.4 ± 6.2	74.5 ± 6.6	0.002
Female, *n* (%)	22(52.4)	45(53.6)	33(58.9)	0.78
Years of education (years), mean ± SD	12.0 ± 3.0	10.5 ± 3.7	9.7 ± 3.4	0.003
MMSE score, mean ± SD	28.6 ± 1.2	25.7 ± 2.3	20.4 ± 3.5	<0.001
MoCA score, mean ± SD	26.4 ± 1.7	22.1 ± 3.0	17.3 ± 3.8	<0.001
Hippocampal volume (mm^3^), mean ± SD	3,550 ± 420	3,120 ± 380	2,620 ± 360	<0.001

Within the thin-slice MRI sub-cohort (*n* = 182), a full-feature model without hippocampal volume was first reconstructed using the same feature combination as Model 4 in the main analysis. This model included demographic and basic clinical characteristics, cognitive assessment scores, conventional imaging markers, and blood-based biomarkers. This model was defined as sub-cohort Model 4. Based on this model, hippocampal volume derived from thin-slice MRI was further added to construct the thin-slice MRI extended model (Model 5). Both models were developed within the same sub-cohort to ensure comparability. Their predictive performances are summarized in Table 8.

**Table 8:** Results of the thin-slice MRI sub-cohort analysis (*n* = 182).

Model	AUC	Sensitivity	Specificity	Brier score
Model 4	0.83 ± 0.03	0.80 ± 0.04	0.79 ± 0.05	0.15
Model 5	0.88 ± 0.02	0.84 ± 0.04	0.85 ± 0.04	0.13

Model 4 refers to the full-feature model reconstructed in the thin-slice MRI sub-cohort using the same feature combination as Model 4 in the main analysis. It was used for comparison with Model 5, which included hippocampal volume. Due to differences in sample sources, the model parameters and predictive performance of sub-cohort Model 4 are not directly comparable with Model 4 reported in Table 5.

The results showed that in the thin-slice MRI sub-cohort, sub-cohort Model 4 already demonstrated good predictive ability for MCI-to-AD conversion. The AUC was 0.83 ± 0.03, with a sensitivity of 0.80 ± 0.04 and a specificity of 0.79 ± 0.05. After inclusion of hippocampal volume, the predictive performance of Model 5 further improved. The AUC increased to 0.88 ± 0.02. Sensitivity and specificity increased to 0.84 ± 0.04 and 0.85 ± 0.04, respectively. The Brier score decreased to 0.13.

## 4 Discussion and conclusion

In recent years, multimodal approaches have been widely applied in studies of the Alzheimer’s disease (AD) continuum. These methods integrate cognitive assessments, imaging features, and biomarker information to improve the accuracy of disease staging and conversion prediction (Di Tella et al., 2021; Khan and Zubair, 2022). However, many previous studies were based on strictly selected research cohorts or relied on cerebrospinal fluid testing and molecular imaging. These approaches involve high cost or invasive procedures. As a result, study populations, diagnostic pathways, and data completeness often differ from real-world clinical settings (Leuzy et al., 2025; Villemagne et al., 2021). In routine clinical practice, available assessment methods vary substantially across patients. The accessibility and completeness of different data modalities are inconsistent. Compared with highly standardized research databases or publicly available cohorts, real-world clinical data show greater complexity and heterogeneity in patient sources, examination pathways, and data completeness. This situation may limit the direct translation of findings from previous studies into routine clinical practice (Sherman et al., 2016). At the same time, real-world data is derived from routine clinical workflows, which more accurately reflects the treatment pathways and clinical characteristics of patients in actual medical settings. Therefore, it has certain advantages in assessing the practical applicability of predictive models. Based on this background, the present study used a real-world clinical cohort and integrated demographic characteristics, neurocognitive assessments, routine imaging indicators, and blood biomarkers to construct a multimodal prediction model for disease stage classification of AD and for predicting the risk of conversion from mild cognitive impairment (MCI) to AD. The results indicated that the multimodal model showed good discriminative ability and stability for both disease stage identification and conversion prediction, and it may have potential application value in real clinical settings.

In pairwise disease stage classification analyses, model discrimination improved progressively as cognitive assessments, imaging features, and blood biomarkers were added. Models integrating multimodal information consistently achieved the best performance across classification tasks. This finding aligns with previous evidence showing that a single modality cannot fully capture the complex pathology of AD (Afridi et al., 2026; Dwivedi et al., 2022). Prior studies reported good performance of models based solely on cognitive tests or structural MRI in distinguishing CN from AD, but markedly reduced accuracy in MCI-related classification tasks (Grueso and Viejo-Sobera, 2021). Similar patterns were observed in the present study. Discrimination between CN and AD was superior to that between MCI and AD, further reflecting the high clinical and biological heterogeneity of the MCI stage. Unlike studies relying on cerebrospinal fluid or PET imaging (Zetterberg and Bendlin, 2021), this study achieved favorable classification performance using routinely available cognitive assessments, semi-quantitative MRI markers, and blood-based biomarkers. These findings suggest that multimodal integration can partially compensate for the limitations of single-modality approaches in real-world clinical environments.

In the analysis of the prediction model for conversion from MCI to AD, the results showed that model performance improved progressively as cognitive assessment results, imaging features, and blood biomarkers were gradually integrated. Compared with previous studies, this research constructed a multimodal prediction model based on real-world clinical data. While maintaining predictive performance, the model also improved accessibility and potential application value in routine clinical practice (Spasov et al., 2019). Similar to earlier reports, cognitive assessments represented the most influential single modality for conversion prediction (Rostamzadeh et al., 2022). The inclusion of imaging markers further improved discrimination, which is in line with evidence supporting the independent value of structural MRI in predicting cognitive decline and dementia risk (Xie et al., 2023). In addition, blood-based biomarkers provided complementary pathological information (Leuzy et al., 2022). Compared with studies relying on cerebrospinal fluid or molecular imaging (Bălașa et al., 2020), the present results indicate that blood Aβ and tau-related markers can improve conversion prediction at the population level without increasing invasiveness. This finding further supports the potential value of blood biomarkers in real-world applications. The results of multivariable Logistic regression analysis showed that cognitive function scores, imaging indicators, and related biomarkers were all associated with the risk of conversion from MCI to AD. These findings partly explain the mechanism underlying the improved performance of the multimodal model and also increase the interpretability of the model. Within the thin-slice MRI sub-cohort, the inclusion of hippocampal volume led to additional performance improvement. This result indicates that even within multimodal models, hippocampal volume provides incremental predictive value. Previous studies have consistently identified hippocampal atrophy as one of the most specific structural imaging markers of AD, often preceding widespread brain atrophy (Chong et al., 2021). Unlike studies that treat hippocampal volume as a core predictor for all participants, the present study did not require this variable in the full cohort. Instead, its enhanced role was clarified through sub-cohort analysis. This approach better reflects real-world clinical practice, where access to high-resolution imaging varies among patients.

Several limitations should be considered in this study. First, this study was based on real-world clinical data from a single center. Although internal validation was performed using five-fold cross-validation and Bootstrap bias correction, an independent external dataset was not available. Therefore, the generalizability of the model across different clinical centers or populations still needs further evaluation in future studies. Moreover, thin-slice MRI analysis was only conducted in a sub-cohort, so the related results should be interpreted with caution.

In summary, this study developed a multimodal prediction model using real-world clinical data. Most of the included features were obtained from routine clinical examinations, which improved the feasibility of applying the model in real clinical settings to some extent. In addition, the discrimination ability, calibration performance, and potential clinical usefulness of the model were systematically evaluated using five-fold cross-validation, calibration curves, and decision curve analysis. Compared with datasets derived from strictly selected research cohorts or public databases, the real-world clinical data used in this study are closer to actual clinical environments in terms of patient composition, examination pathways, and data completeness. This feature highlights the advantage of this data source in reflecting real clinical scenarios. Importantly, the goal of this study was not to construct an optimal prediction model based on invasive or high-cost examinations. Instead, it aimed to assess the practical value of multimodal integration using routinely available information in real-world clinical settings. Consequently, the results are mainly applicable to risk assessment and stratified management in routine care rather than replacing research paradigms centered on molecular imaging or cerebrospinal fluid biomarkers. Future studies should validate these models in multicenter prospective cohorts. Further research is also needed to explore the incremental value of high-precision imaging or molecular markers across different risk strata. Such efforts may help achieve a balanced optimization between predictive performance and clinical feasibility.

## Data Availability

The raw data supporting the conclusions of this article will be made available by the authors, without undue reservation.
